# Long Term Results of Reduction Ascending Aortoplasty

**DOI:** 10.3390/life12101526

**Published:** 2022-09-30

**Authors:** Cinzia Trumello, Ilaria Giambuzzi, Marta Bargagna, Kevin Tavana, Arturo Bisogno, Guido Ascione, Mariachiara Calabrese, Alessandro Castiglioni, Ottavio Alfieri, Michele De Bonis

**Affiliations:** 1 IRCCS San Raffaele Scientific Institute, 20100 Milan, Italy; 2 Department of Cardiac Surgery, Vita-Salute San Raffaele University, 20100 Milan, Italy

**Keywords:** ascending aorta dilation, reduction ascending aortoplasty, ascending aorta ectasia

## Abstract

The aim of this retrospective study is to show medium-long-term results in terms of cardiac death and aortic events in patients undergoing reduction ascending aortoplasty between 1997 and 2009 in our hospital. The Fine and Grey model for competing risk analysis was performed for time to cardiac death, with non-cardiac death as the competing risk, and time to recurrence of both re-dilation (aortic diameter > 45 mm) and re-operation with overall death as the competing risk. Paired *t*-test was used to evaluate the change in aortic diameter from the post-operative values to follow-up. The population included 142 patients. The mean pre-operative aortic diameter and the diameter at follow-up were respectively 46.5 ± 5.11 mm vs. 41.4 ± 5.55 mm (*p*-value < 0.001). At a mean follow-up of 11.6 ± 4.15 years, 11 patients (7.7%) required re-operation on the ascending aorta. At 16 years, the CIF of aortic-related events was 29.4 ± 7.2%; the freedom from cardiac death was 89.2 ± 3.7%. Ten patients (7%) died from cardiac causes but no one was aortic-related. The Fine and Grey analysis did not identify any significant predictors. This procedure is safe but might be justified only in high-risk patients or in those with advanced age/short life expectancy.

## 1. Introduction

In adult patients, the aortic diameters of ascending aorta do not normally exceed 40 mm and then gradually decrease. A lot of factors including age, gender, blood pressure, and body characteristics as well as height, weight, and body surface area (BSA), influence its diameter [1,2,3,4,5,6].

It conveys all the blood expelled from the heart first to the coronary arteries, then to the head and neck area, and after that, to the rest of the body.

Nevertheless, the aorta is not a passive tube, because it has pressure-responsive receptors located in its wall that play an important role in the control of systemic vascular resistance and heart rate [7]. The elasticity of the aortic wall performs the Windkessel function as a ‘second pump’ during diastole, which is of great importance for central e peripheral perfusion.

Ascending aortic aneurysms (AAA) are generally treated with resection and replacement of the aneurysmatic segment because of the risk of dissection and/or rupture. Current guidelines [8,9] on the treatment of AAA state that surgery should be considered in patients with maximal ascending aortic diameters of 45 mm for patients with connective tissue diseases with risk factors, 50 mm for patients with bicuspid valve with risk factors, and 55 mm for other patients with no elastopathy. Lower thresholds for intervention may be considered according to BSA in patients of small stature or in the case of rapid progression, aortic valve regurgitation, planned pregnancy, and patient’s preference. Class IIa (weight of evidence/opinion is in favor of usefulness/efficacy), level of evidence C (Consensus of opinion of the experts and/or small studies, retrospective studies, registries).

Nevertheless, replacement of AAA can be considered also in patients with a maximum diameter ≥45 mm in case of concomitant cardiac surgery [10].

However, the replacement of AAA is associated with longer cardio-pulmonary bypass (CPB) time, with mortality rates up to 10% [11] even if in more recent years mortality rates have been lowered [12,13], and the indications in moderate AAA remain debatable [14,15] even if untreated patients with AAA have shown a higher risk of dissection [16]. Nevertheless, also on this point, there is an ongoing debate on which is the real threshold of risk for aortic dissection, indeed some authors [17,18] found a high risk of aortic dissection at a diameter lower than the one proposed in the guidelines. Therefore, many surgeons try to reduce the aortic diameter also when it does not reach the threshold for replacement. Over the years, many different surgical options have been proposed to reduce the diameter of the AAA without replacing it, such as reduction ascending aortoplasty (RAA) with or without external wrapping [19,20,21,22,23,24,25]. In these procedures, the aorta is not resected but is remodeled externally. Reduction ascending aortoplasty is less invasive than a replacement of AAA, it results to be safe and reproducible [19,20,21,22,23,24,25]. Moreover, this procedure is associated with lower rates of transfusion and shorter ICU stay [26]. At present time, it is still unclear the risk of re-dilation, dissection, and re-operation in the long term; for this reason, the procedure is limited to patients who are expected to have long CPB times and higher surgical risks.

The aim of our study is to report the medium- and long-term results of RAA without external wrapping in our population and to investigate any evidence of predictors of aortic-related events, such as the aortic re-dilation or/and re-intervention on the ascending aorta, and cardiac death.

## 2. Materials and Methods

The study was approved by the local Ethical Committee (registry number 79/INT/2018, 4 June 2018). The need for informed consent was waived because of the retrospective nature of the study. All patients who underwent RAA concomitant to other cardiac procedures between 1997 and 2009 were included. Preoperative and postoperative data were prospectively entered into a dedicated database. Clinical and echocardiographic follow-ups were collected through phone calls, institutional outpatient clinics, and by direct contact to the referral cardiologist.

RAA was performed as a concomitant surgery when the ascending aorta did not meet the criteria of the guidelines for replacement of ascending aorta or when the diameter was ≥45 mm and/or the quality of the aortic tissue was poor and/or when the surgeon thought that the replacement would be at high risk.

### 2.1. Surgery

**Figure 1:**
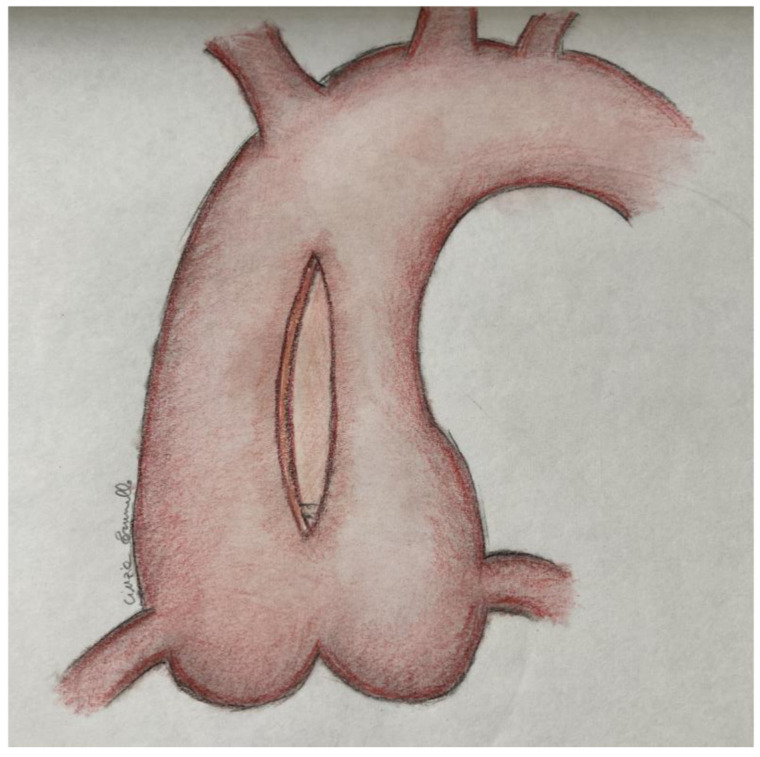
Longitudinal incision on the aorta.

**Figure 2:**
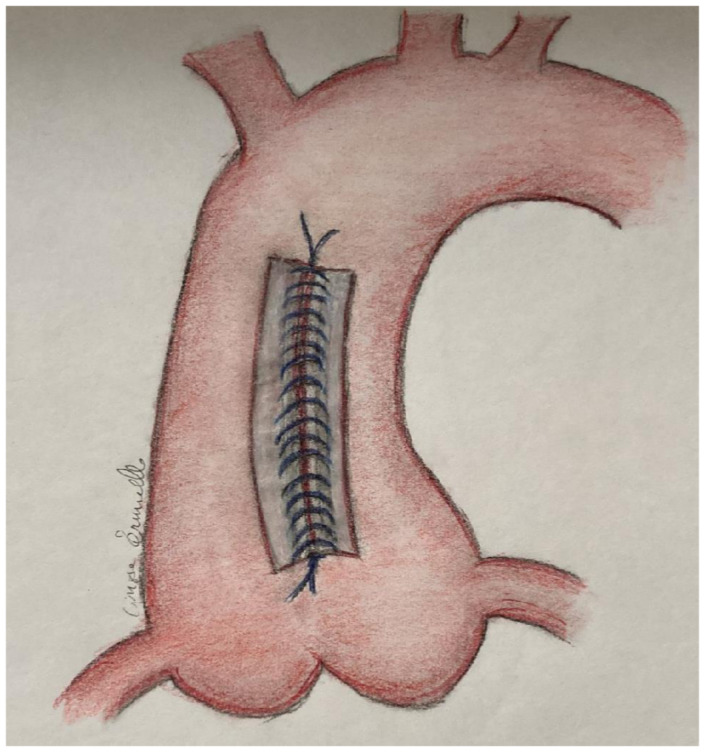
Closure of the aorta with double running suture reinforced with Teflon.

All patients underwent median longitudinal sternotomy, the aortic cannula was inserted in the proximal aortic arch and either a right atrial or a bicaval CPB was established, according to the concomitant procedure scheduled. After aortic cross-clamping, antegrade cardioplegia solution was administered, in moderate hypothermia, in the aortic root and/or selectively in the coronary ostia to obtain cardioplegic arrest. The concomitant aortic valve surgeries were carried out through a longitudinal aortotomy from a few millimeters below the aortic clamp towards the commissure between the non-coronary sinus and the right coronary sinus (Figure 1). After the aortic valve procedure was completed, the aortotomy was sutured with 4-0 Prolene double-running sutures reinforced with two Teflon stripes, to ensure an efficacious reduction in the diameter (Figure 2). Concomitant procedures (coronary artery bypass surgery and/or mitral valve surgery) were performed. When the aortic valve was not replaced, coronary artery bypass surgery was performed first, followed by RAA with the same technique previously described. Afterwards, the patient was weaned from cardiopulmonary bypass and an echocardiography check was performed before the routine closure of the chest and transfer to the intensive care unit.

### 2.2. Statistical Analysis

Preoperative and postoperative data were prospectively entered into a dedicated database. Statistical analyses were performed using Stata software version 13. Data are reported as the mean ± standard deviation (SD) or median with interquartile range (IQR) for continuous variables. Categorical variables were expressed as numbers and percentages and analyzed by means of a two-tailed 𝜒2 test. Kaplan–Meier survival curves were generated to analyze long-term survival. A multivariable Cox regression analysis was performed to identify correlates of mortality. For competing risk, the cumulative incidence function (CIF) was computed for time to cardiac death with non-cardiac death as competing risk, for time to reoperation and re-dilatation (>42 cm) with death as competing risk. The Fine and Grey model for competing risk was employed in the analysis for the assessment of predictors of aortic-related events, cardiac death, and reoperation. A *p*-value of less than 0.05 was used to define statistical significance.

Paired T-test was used to evaluate the change in aortic diameter from the post-operative to the follow-up values.

## 3. Results

Between 1997 and 2009, a total of 142 consecutive patients underwent cardiac surgery with concomitant RAA. Among them, there were 42 (30%) female patients, and the mean age was 64.4 ± 10.46. No patient had a known genetic disease (i.e., Marfan syndrome). The pre-operative characteristics are extensively listed in Table 1 and expressed either as mean and standard deviation or median and interquartile range.

The pre-operative mean aortic diameter was 46.5 ± 5.11 mm. The most frequent indication for surgery was an aortic valve disease, which was either a stenosis, regurgitation, or a combination of the two, respectively, in 43 (30.3%), 71 (50%), and 23 (16.2%) patients, 5 patients (3.52%) underwent solely a coronary artery bypass grafting. The anatomy of the aortic valve is described in Table 2.

Aortic valve replacement was performed on 137 patients (96%), using either a mechanical or biological prosthesis. In 1 patient (0.7%) a REDO intervention was performed to replace a previously implanted prosthesis. The median CPB time was 73 min (IQR 63–87 min) and the median aortic cross-clamping time was 54 min (IQR 45–68 min). Post-operative complications are listed in Table 3. Patients were dismissed from the hospital with standard cardiovascular therapy, including beta-blockers and angiotensin-receptor blockers (ARBs).

The diameter of the aorta was evaluated at the pre-discharge echocardiogram and the mean diameter was significantly reduced compared with the pre-operative value: 36.5 ± 3.61 mm vs. 46.5 ± 5.11 mm (*p*-value < 0.001).

### 3.1. Follow Up

**Figure 3:**
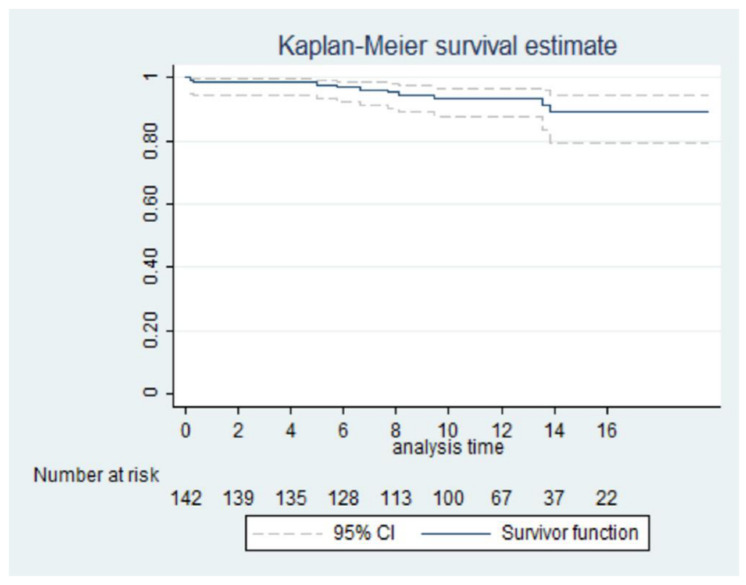
Kaplan–Meier, overall survival.

The follow-up was 100% complete and the mean length was 11.6 ± 4.15 years. The longest follow-up time was 19.92 years. The mean diameter of ascending aorta at follow-up was 41.1 ± 6.19 mm, significantly higher than the post-operative diameter of 36.5 ± 3.61 mm (*p*-value < 0.001) but, anyway, it remains significantly reduced compared with the preoperative value of 46.5 ± 5.11 mm (*p*-value < 0.001). The Kaplan–Meier curve of the overall survival is shown in Figure 3.

### 3.2. Cardiac Death

**Figure 4:**
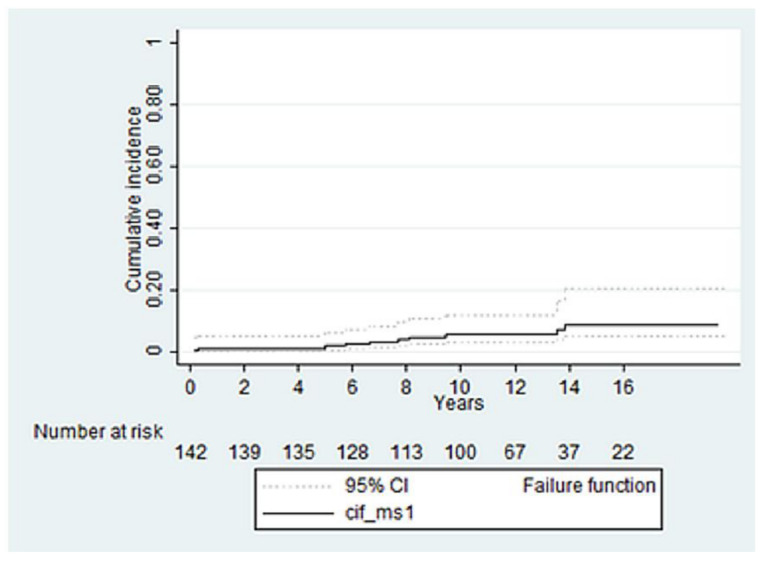
CIF of cardiac death with non-cardiac death as competing risk at 16 years.

The CIF of cardiac death with non-cardiac death as competing risk at 16 years was 8.7 ± 2.8 (Figure 4).

The associated Fine and Grey analysis found as a protective factor a higher ejection fraction at time of surgery (SHR 0.87 CI 0.81–0.94, *p*-value < 0.001); on the contrary, as a risk factor, a higher BSA at time of intervention (SHR 1.06 CI 1.03–1.01, *p*-value < 0.001); the other tested variables (age, sex, ascending aortic diameter >46 mm and aortic disease) were not statistically significant.

### 3.3. Ascending Aorta Re-Intervention

**Figure 5:**
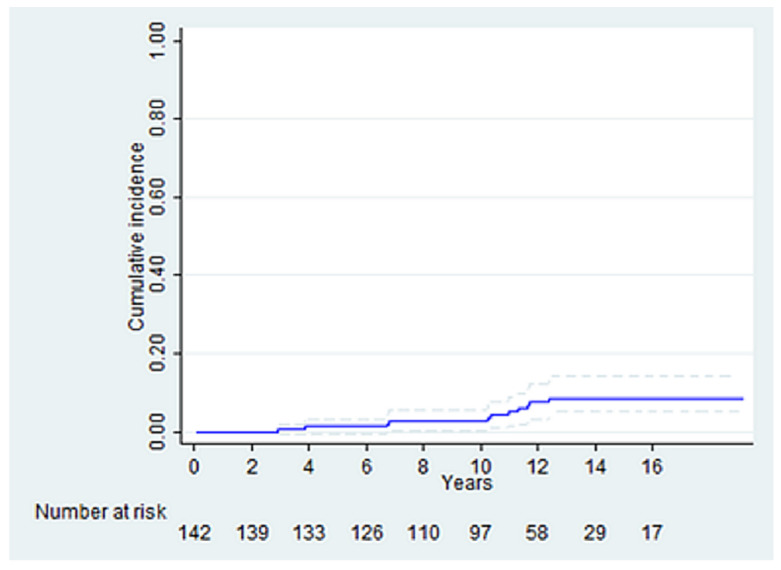
CIF of aortic re-intervention with death as competing risk at 16 years.

At 16 years, the CIF of re-intervention with death as competing risk at 16 years was 8.8 ± 2.6% (Figure 5).

It did not have any significant variables in the Fine and Grey analysis.

### 3.4. Aortic-Related Events

**Figure 6:**
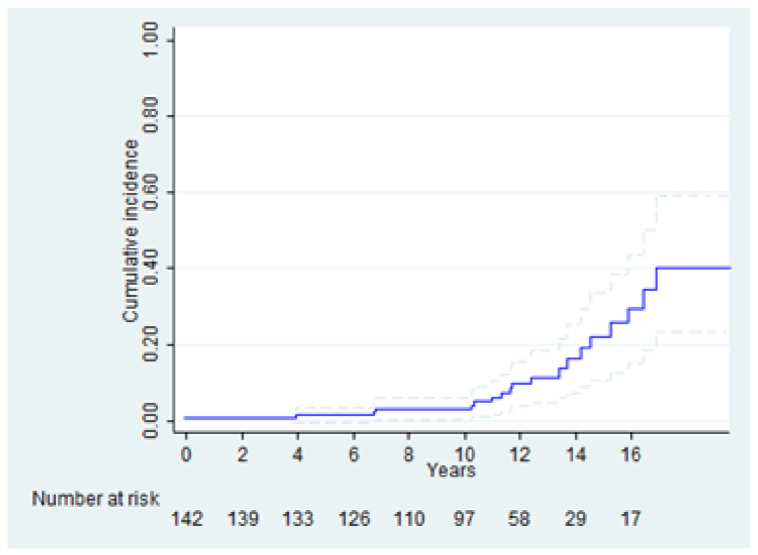
CIF of aortic-related events (including aortic re-dilation with a diameter >45 mm) and/or re-intervention on the ascending aorta with death as competing risk at 16 years.

Regarding aortic-related events, such as the aortic re-dilation (considered as aortic diameter >45 mm) or/and re-intervention on the ascending aorta, the CIF with death as competing risk, reported the risk of 29.4 ± 0.72% at 16 years (Figure 6), without any statistically significant tested variables.

The difference between the post-operative value of aortic diameter and the follow-up diameter, respectively 36.0 ± 1.6 mm vs. 41.4 ± 5.55 mm was in fact significantly different (*p*-value < 0.001).

We found no differences among the rate of aortic-related events depending on valve anatomy, but with the Fine and Grey model, younger age had a higher risk of being re-operated later in life (OR 0.93, CI 0.87–0.99, *p*-value 0.03). Moreover, because of the re-dilation of the ascending aorta, in our series, 11 patients (9%) needed ascending aorta surgery (either isolated or combined to re-replacement of degenerated bioprosthesis).

## 4. Discussion

Treatment of moderate dilatation of ascending aorta, in patients with a complex scheduled operation or with high surgical risk or short life expectancy, remains a topic of debate [25,26,27,28,29,30,31]. The literature accounts for some short/mid-term experiences in which Bauer et al. [32] reported a survival rate of 94% at 5 years; Shen Liu et al. [33] described a freedom from death at 3 years of 98% and Polvani et al. [34] reported an overall survival estimate of 89.3% ± 5.9% at 6 years being in line with other published literature. While the literature on ascending aorta replacement shows high rates of perioperative mortality and morbidity [35,36], our study shows mid-term and long-term results comparable to those reported by other studies and reports the long-term evidence up to 16 years after RAA concomitant to other procedures. After 16 years, 88% of patients were alive and the risk of cardiac death, according to the CIF, was less than 9%. The CIF of cardiac death we reported is 12%, without any aortic-related events.

As mentioned before, different techniques of RAA have been described over the years, with or without external wrapping [19,20,21,22,23,24,25]. When external wrapping is performed, a synthetic fold is often used to enforce the external support [20,21,22,23]. It has been described that unfixed wrap may cause severe aortic wall degeneration with thinning of the ascending aorta [27,37]; moreover, Neri et al. [26] indicated the external banding as a potential cause of the aortic wall degeneration, as well as Walker and colleagues [28], who suggested that unsupported aortoplasty may preserve the Windkessel function of the ascending aorta. All of this evidence makes the unforced RAA more attractive than external wrapping. Indeed, also replacement of the ascending aorta impacts the elasto-mechanical properties of the ascending aorta: the “compliance mismatch”, as Cristiano Spadaccio et al. [38] have called it. Spadaccio et al. [38] performed a review of the literature and they not only found the same loss of the Windkessel effect in the case of ascending aorta replacement but also published literature demonstrating that the loss of the elasto-mechanical properties of the ascending aorta had a local effect (stress on suture lines, aortic valve dysfunction) and systemic effect (vascular inflammation). We agree with the authors that further research is needed to find an adequate substitute for Dacron graft, as it might be of particular importance for very young or syndromic patients.

The short median CPB time and the median aortic cross-clamping time clearly demonstrate a low impact on the surgical risk; furthermore, RAA is associated with a lower need for transfusions and shorter ICU stay [29,39]. On the other hand, our results on aortic-related events, such as aortic re-dilation or/and re-intervention on the ascending aorta, show a CIF risk of 30% at 16 years related to the statistically significant increase in the aortic diameter at follow-up, compared with the post-operative value.

**Figure 7:**
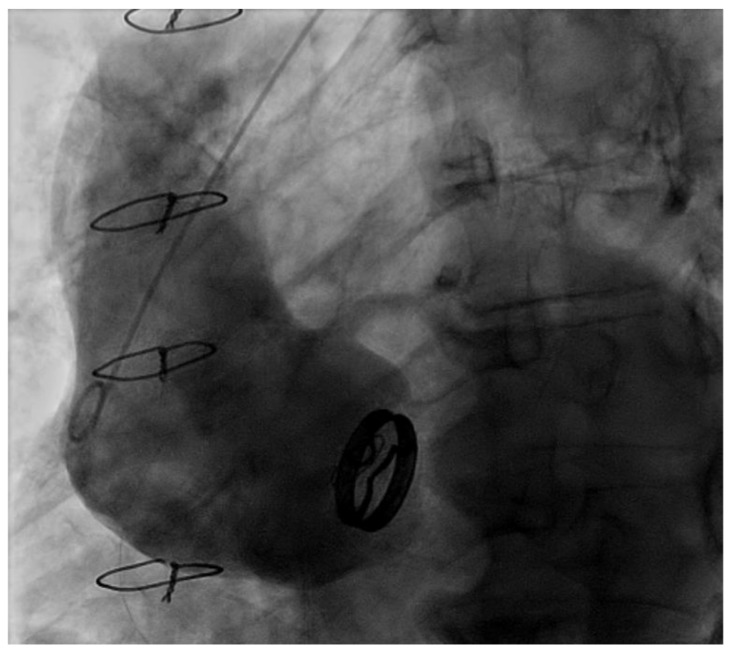
Aortography of ascending aorta re-dilation after RAA.

Some studies suggest some degree of association to the risk of re-dilation or re-operation, depending on valve anatomy, as a bicuspid aortic valve has a higher risk [40,41,42]. In our study, no differences were found in the rate of aortic-related events depending on valve anatomy, but a direct relationship between young age and risk of re-operation over the years. Among the 11 patients (9%) of our series that needed ascending aorta surgery, 3 (27.3%) had bioprosthesis degeneration as the primary indication of the reintervention; consequently, aortic replacement became necessary to perform the REDO on the aortic valve (Figure 7).

This result prompts some thoughts on the indication for AAA surgery. Current guidelines [8,9,10] suggest replacing AAA when the diameter exceeds 45 mm in the case of concomitant surgery. Nevertheless, replacement of AAA might raise the surgical risk too much, especially in fragile patients; therefore, there is a need for low-risk surgery, such as RAA, to reduce the ascending aorta diameter. However, in our sample of patients, there was a high risk of having aortic-related events at 16 years, despite having a relatively small diameter. There should probably be a re-evaluation of the current guidelines because the diameter of the ascending aorta might not be enough to pose an indication for surgery. Indeed, the threshold for surgery is based on the findings at the time of aortic dissection [43], which are different from the pre-dissection dimensions. Many authors suggest using scores that integrate the aortic diameter with its length/patients’ BSA and so on [44,45]. Moreover, also genetic testing [46] should be taken into account when deciding whether or not a patient should undergo a more aggressive surgery on the ascending aorta. Further studies are needed to delineate which subset of patients would benefit the most from a replacement of AAA at a lower threshold.

Moreover, the importance of ascending aorta re-dilation becomes of pivotal importance in young patients who might be future TAVI candidates. Patients with re-dilated ascending aorta have greater surgical risk during TAVI [47], and the procedure might even be contro-indicated, leaving high-risk patients no choice. Therefore, when planning a surgery on border-line AAA, the surgeon should keep in mind that the patient might need a further procedure on the aortic valve, which is possible in case of replacement of ascending aorta, but much more risky if the RAA leads to a re-dilation.

Therefore, RAA has a high rate of mid- and long-term aortic-related events, but in selected patients might ease the trauma inflicted by the surgery, accepting it as an alternative in a selected group of patients. Nevertheless, experienced surgeons in the context of the heart team should decide if RAA might be feasible. In the future, it is desirable that specialized centers would offer Aortic Team (as some high-volume centers are already doing), so that every patient could receive a personalized surgery.

### Limitations

Our study has several limitations related primarily to the retrospective nature of the study itself. Secondly, the lack of intra-operative data such as the amount of blood loss and the need for blood transfusions could support the choice of this procedure in patients at greater risk. No histologic exams were performed. The echocardiographic follow-up does not represent the diagnostic gold standard for aortic dilatation, but it was almost impossible, both for clinical and organizational reasons, to perform a CT scan on all patients; moreover, data on ascending aorta diameter were not always present. All these limitations and the lack of data in the literature suggest how this field of research would benefit from further scientific investigation.

## 5. Conclusions

The technique of RAA brings an immediate intraoperative advantage in terms of surgical risks. However, the long-term results show that beyond 10 years 30% of the patients develop significant re-dilatation of the aorta, and part of them require a redo-surgery. Nowadays, the progressive increase in TAVI procedures has a strong impact on cardiac surgery activity and therefore must be taken into consideration before applying this type of intervention and its impact on future therapeutic options available to the patient.

It underlines the need for a careful balance of risks and benefits in the decision-making process of patient selection for this technique whose results might be justified only in high-risk patients or in those with advanced age/short life expectancy.

## Figures and Tables

**Table 1:** Pre-operative characteristics.

Variable	*N* (%)
Age	64.4 ± 10.46
Female sex	42 (30)
BMI	25.3 [22.8, 27.2]
BSA	1.82 [1.69, 1.93]
Smoking	18 (13)
Hypertension	90 (63)
Diabetes	12 (8.5)
Chronic renal failure	10 (7.0)
**Echocardiographic parameters**	
Ascending aorta	46.5 ± 5.11
Ejection fraction	56 [47, 60]
End diastolic diameter	57 [50, 65]
**Diagnosis**	
Aortic stenosis	43 (30.3)
Aortic regurgitation	71 (50)
Mixed aortic disease	23 (16.2)
Coronaropathy	5 (3.5)

**Table 2:** Anatomy of the aortic valve.

Aortic Valve	*N* (%)
Bicuspid	35 (25)
Tricuspid	83 (58)
Prosthesis	1 (0.7)
Unknown	23 (16.3)

**Table 3:** Post-operative complications.

Variable	*N* (%)
Arrhythmias	33 (23)
Neurologic events	1 (0.7)
Pericardial effusion	3 (2.1)
Surgical revisions	3 (2.1)
PM implant	4 (2.8)
Acute kidney failure	6 (4.2)
Blood transfusions	16 (11)
Low cardiac output syndrome	3 (2.1)
Respiratory failure	4 (2.8)
Others	5 (3.5)

Arrhythmias: Atrial fibrillation, atrial flutter. Neurologic events: Strokes and transitory ischemic attack. Respiratory failure: Prolonged mechanical ventilation (>48 h) or need of NIMV or re-intubation. Others: Sepsis, surgical site infection, delirium.

## Data Availability

The data presented in this study are available on request from the corresponding author. The data are not publicly available due to privacy reasons.
